# Can nutritional interventions change major clinical outcomes?

**DOI:** 10.1186/s13054-018-2085-y

**Published:** 2018-06-08

**Authors:** Sho Horikita, Masamitsu Sanui, Alan K. Lefor

**Affiliations:** 1 Department of Anesthesiology and Critical Care Medicine, Jichi Medical University Saitama Medical Center, 1-847 Amanumacho Omiya, Saitama, Saitama 330-8503 Japan; 2 Department of Surgery, Jichi Medical University, 3311-1 Yakushiji, Shimotsuke-shi, Tochigi, 329-0498 Japan

**Keywords:** Enteral nutrition, Parenteral nutrition, Critically ill, Permissive underfeeding

In the NUTRIREA-2 trial [1] recently published in the *Lancet*, the authors assessed whether early enteral nutrition (EN) decreases mortality compared with early parenteral nutrition (PN) in patients undergoing invasive mechanical ventilation and vasopressor support for shock, with normo-caloric targets for both routes of support. As the primary endpoint, mortality on day 28 was comparable between the groups. However, we believe that nutritional protocols for both study groups deviated from current standards of nutritional therapy for critically ill patients, which makes interpretation of the results difficult.

There are no clear data showing that EN targeting full caloric requirements immediately after admission is superior to permissive hypocaloric EN [2, 3]. Recent large randomized controlled trials (RCTs) even suggest that full caloric EN has some disadvantages over hypocaloric EN, including gastrointestinal intolerance [2]. The latest Society of Critical Care Medicine and American Society for Parenteral and Enteral Nutrition guidelines [4] suggest that EN is the preferred nutritional route for the majority of critically ill patients. Therefore, there is no clear justification for patients in refractory shock to receive full-caloric EN as in the NUTRIREA-2 trial [1], although these unstable patients may tolerate trophic feeding or underfeeding. In the real world, critical care staff would take significant advantage of less gastrointestinal complications along with non-inferiority of hypocaloric to normocaloric EN.

Also, there are no definite data showing that early PN beginning within a few days of ICU admission, either supplemental or exclusive PN to meet full-caloric goals, has a significant clinical benefit. In the EPaNIC trial [5], early PN supplementation to hypocaloric EN for full caloric targets did not improve mortality in critically ill patients, but did increase the rate of infections. Therefore, full-caloric PN is not the current standard nutritional therapy for critically ill patients.

For unstable patients requiring vasopressors and mechanical ventilation, we believe that trophic EN is the first-line nutritional therapy. Based on the fact that none of the EN or PN nutritional protocols [1–3, 5] showed a benefit of nutritional therapy on clinically important outcomes in critically ill patients, additional efforts to investigate a particular nutritional therapy to improve clinical outcomes during the acute phase may not be advocated. It is rather reasonable to investigate a nutritional therapy to do the least harm. We need to appreciate the idea that nutrition does not give strength to patients, but can potentially do harm to patients.

## Abbreviations

EN: Enteral nutrition
PN: Parenteral nutrition
RCT: Randomized controlled trial
